# TAP1, a potential immune-related prognosis biomarker with functional significance in uveal melanoma

**DOI:** 10.1186/s12885-023-10527-9

**Published:** 2023-02-11

**Authors:** Ru Zhu, Yu-Ting Chen, Bo-Wen Wang, Ya-Yan You, Xing-Hua Wang, Hua-Tao Xie, Fa-Gang Jiang, Ming-Chang Zhang

**Affiliations:** 1 Department of Ophthalmology, Union Hospital, Tongji Medical College, Huazhong University of Science and Technology, Wuhan, 430022 China; 2 Cancer Center, Union Hospital, Tongji Medical College, Huazhong University of Science and Technology, Wuhan, 430022 China

**Keywords:** TAP1, Uveal melanoma, Prognostic biomarkers, Immunomodulation, Metastasis

## Abstract

**Background:**

TAP1 is an immunomodulation-related protein that plays different roles in various malignancies. This study investigated the transcriptional expression profile of TAP1 in uveal melanoma (UVM), revealed its potential biological interaction network, and determined its prognostic value.

**Methods:**

CIBERSORT and ESTIMATE bioinformatic methods were used on data sourced from The Cancer Genome Atlas database (TCGA) to determine the correlation between TAP1 expression, UVM prognosis, biological characteristics, and immune infiltration. Gene set enrichment analysis (GSEA) was used to discover the signaling pathways associated with TAP1, while STRING database and CytoHubba were used to construct protein–protein interaction (PPI) and competing endogenous RNA (ceRNA) networks, respectively. An overall survival (OS) prognostic model was constructed to test the predictive efficacy of TAP1, and its effect on the in vitro proliferation activity and metastatic potential of UVM cell line C918 cells was verified by RNA interference.

**Results:**

There was a clear association between TAP1 expression and UVM patient prognosis. Upregulated TAP1 was strongly associated with a shorter survival time, higher likelihood of metastasis, and higher mortality outcomes. According to GSEA analysis, various immunity-related signaling pathways such as primary immunodeficiency were enriched in the presence of elevated TAP1 expression. A PPI network and a ceRNA network were constructed to show the interactions among mRNAs, miRNAs, and lncRNAs. Furthermore, TAP1 expression showed a significant positive correlation with immunoscore, stromal score, CD8+ T cells, and dendritic cells, whereas the correlation with B cells and neutrophils was negative. The Cox regression model and calibration plots confirmed a strong agreement between the estimated OS and actual observed patient values. In vitro silencing of TAP1 expression in C918 cells significantly inhibited cell proliferation and metastasis.

**Conclusions:**

This study is the first to demonstrate that TAP1 expression is positively correlated with clinicopathological factors and poor prognosis in UVM. In vitro experiments also verified that TAP1 is associated with C918 cell proliferation, apoptosis, and metastasis. These results suggest that TAP1 may function as an oncogene, prognostic marker, and importantly, as a novel therapeutic target in patients with UVM.

**Supplementary Information:**

The online version contains supplementary material available at 10.1186/s12885-023-10527-9.

## Background

Uveal melanoma (UVM) is the most common primary intraocular malignancy in adults (85%) and is highly malignant, invasive, and extremely metastatic with a poor prognosis [1, 2]. In current practice, the first-line treatment of primary uveal melanoma is local ocular treatment, including plaque brachytherapy, local resection, and enucleation [3]. Over the last 5 years, novel prognostic biomarkers have been identified, including histone deacetylase HDAC-2 [4], DNA damage response protein ATR [5], and genes of the NF-κB pathway [6]. Although the treatments and prognostic biomarkers have improved over the past decade, the tumor-related mortality rate has remained unchanged and most metastasis cases are fatal within 1 year [7, 8]. Therefore, there is an urgent need to improve our understanding of the molecular and cellular biology of UVM to aid in the development of new prognostic biomarkers and personalized treatment regimens.

Transporter associated with antigen processing 1 (TAP1) is a membrane-bound protein consisting of two membrane-spanning domains that belongs to the ATP-binding cassette (ABC) transporter superfamily [9]. TAP1 is involved in antigen transport from the cytoplasm to the endoplasmic reticulum, binds to major histocompatibility complex (MHC) class I molecules, forms the molecular scaffold for the final stage of MHC class I folding, and then presents MHC I to immune cells, thereby serving as a target for tumor cells [10]. Recent studies have shown that TAP1 is closely associated with various tumors and has the potential to become a novel tumor-targeting drug [11, 12]. Although high TAP1 expression might, in theory, make tumor cells more susceptible to cytotoxic T cell killing [13], it might reduce their susceptibility to natural killer (NK) cell–mediated lysis [14]. Therefore, there is no consistent relationship between TAP1 expression and patient prognosis for different cancer types.

No previous study has investigated TAP1 expression in uveal melanoma. The aim of this study is to investigate the association between TAP1 expression, disease prognosis, and biological score in patients with UVM, and whether TAP1 plays a role in the UVM immune microenvironment. We performed in vitro experiments to verify the effects of TAP1 on the proliferation and migration of UVM cells. These findings may further improve the accuracy of prognostic prediction of UVM and present a potential therapeutic target for this type of cancer.

## Materials and methods

### Data download

The gene expression data (FPKM values) from the RNA sequencing of 80 patients with UVM were downloaded from the official website of The Cancer Genome Atlas (TCGA) Genomic Data Commons (GDC) (https://portal.gdc.cancer.gov/) and divided into mRNA and long noncoding RNA (lncRNA) expression. The clinicopathological features and prognosis of the individual patients with UVM, such as sex, age, and stage, were downloaded from the University of California, Santa Cruz (UCSC) website (http://xena.ucsc.edu/). The specific clinical information on these patients is presented in Table 1. Moreover, the gene expression data of GSE221381 [15] and clinicopathological features of the patients were downloaded from the Gene Expression Omnibus (GEO) database as the validation dataset. The data originated from *Homo sapiens* samples and were generated on the GPL570[HG-U133_Plus_2] Affymetrix Human Genome U133 Plus 2.0 Array platform, including 63 UVM tissue samples. We also downloaded the gene expression data of GSE84976 [16] and clinicopathological features of the patients GEO database. The data originated from *Homo sapiens* samples and were generated on the GPL10558 Illumina HumanHT-12 V4.0 expression beadchip Array platform, including 28 UVM tissue samples. as a validation dataset.

**Table 1:** The UVM patient database from TCGA

Variables	All patients (*n* = 80)	Low expression (*n* = 40)	High expression (n = 40)	*P* value
Gender				0.822
Female	35 (43.8%)	17 (42.5%)	18 (45.0%)	
Male	45 (56.2%)	23 (57.5%)	22 (55.0%)	
Age				0.653
<60	36 (45.0%)	17 (42.5%)	19 (47.5%)	
≥ 60	44 (55.0%)	23 (57.5%)	21 (52.5%)	
Pathologic stage				0.025*
I & II	36 (45.0%)	23 (57.5%)	13 (32.5%)	
III & IV	44 (55.0%)	17 (42.5%)	27 (67.5%)	
Histological type				0.05*
Epithelioid Cell	13 (16.3%)	3 (7.5%)	10 (25.0%)	
Mixed	37 (46.2%)	18 (45.0%)	19 (47.5%)	
Spindle Cell	30 (37.5%)	19 (47.5%)	11 (27.5%)	
Type				< 0.001***
metastatic	19 (23.8%)	5 (12.5%)	14 (35.0%)	
non-metastatic	61 (76.3%)	35 (87.5%)	26 (65.0%)	
Statue				0.026*
Alive	57 (71.3%)	33 (82.5%)	24 (60.0%)	
Death	23 (28.7%)	7 (17.5%)	16 (40.0%)	

### Differentially expressed genes (DEGs)

To analyze the *TAP1* gene expression in patients with UVM, patients’ tumor samples in TCGA and GEO databases were divided into high- and low-expression groups based on median value of *TAP1* gene expression. Differentially expressed genes (DEGs) between the two groups were analyzed using the *limma* package of R [17], where log fold change (logFC) > 0.5 and adjusted *p*-value < 0.05 were set as the thresholds for statistical significance. The *VennDiagram* package for R [18] was used to select specific DEGs that were common between the two datasets. The results of the difference analysis were presented in the form of a volcano plot.

### Functional enrichment analysis and gene set enrichment analysis (GSEA)

Gene ontology (GO) analysis is a standard method for conducting large-scale functional enrichment studies involving functions categorized under biological processes, molecular functions, and cellular components. The Kyoto Encyclopedia of Genes and Genomes (KEGG) is a widely used database for storing information on genomes, biological pathways, diseases, and drugs [19]. Signature genes were analyzed for functional and pathway enrichment and annotated based on GO and KEGG databases using the *clusterProfiler* R [20] software package, and a cut-off FDR value of < 0.05 was considered statistically significant. To investigate differences in biological processes among the different groups, we performed GSEA on the gene expression profiling dataset of patients with UVM. GSEA is a computational method for analyzing whether a particular gene set is statistically different between two biological states and is usually used to estimate changes in pathway and biological process activity [21] in samples of expression datasets. The “c2.cp.kegg.v6.2.-symbols” [21] gene set was downloaded from the MSigDB database for GSEA analysis and an adjusted *p*-value > 0.05 was considered statistically significant. Signature genes of relevant pathways were downloaded from the GeneCard database, and the ssGSEA analysis algorithm was used to calculate the enrichment scores of each patient in different pathways.

### The tumor immune estimation resource (TIMER) database analysis and comparison of immune-related scores between the two groups

TIMER database (https://cistrome.shinyapps.io/timer/) is an online analytical tool for comprehensive analysis of tumor-infiltrating immune cells [22]. The TIMER algorithm enables users to estimate the composition of six immune-infiltrating cell subsets, namely, B cells, CD4+ T cells, CD8+ T cells, macrophages, neutrophils, and dendritic cells (DCs) in different tumors. We analyzed the correlation between *TAP1* gene expression, mutation, and immune cell infiltration using the TIMER database. ESTIMATE [23] is an algorithm that quantifies immune activity in a particular tumor sample based on gene expression profiles. We assessed the immune activity of each tumor and its stromal score using the R package *estimate* [23] the immune cell infiltration levels between the two groups of samples were compared using the Mann-Whitney U test.

### Construction and validation of clinical prediction models

To further evaluate the influence of *TAP1* gene expression combined with clinicopathological features on patient prognosis, univariate and multivariate Cox analysis risk scores were used in combination with clinicopathological features to independently predict overall survival (OS), and the corresponding indicators were incorporated into the model to construct a clinical prediction nomogram. To quantify the discrimination performance, Harrell’s concordance index was measured. A calibration curve was generated to assess the performance of the nomogram by comparing its predicted values with the observed actual survival rates.

### Construction of protein–protein interaction (PPI) network and screening of hub genes

In this study, we used the Search Tool for the Retrieval of Interacting Genes/Proteins (STRING) online tool, which was designed to predict protein-protein interactions, to construct a PPI network of selected genes. Using the STRING database, genes with scores greater than 0.4 were selected to construct the network model visualized by Cytoscape (v3.7.2) [24]. It has been reported that in co-expression networks, the Maximal Clique Centrality (MCC) algorithm is the most efficient method for finding nodes in a set. The MCC of each node was calculated using the CytoHubba plugin [25] in Cytoscape, and genes with the top eight MCC values were selected as hub genes.

### Construction of a competing endogenous RNA (ceRNA) network based on mRNA-miRNA-lncRNA

Before analyzing the basic statistics, lncRNA–miRNA interaction information was downloaded from the miRcode database, and the information on miRNA–mRNA interactions was downloaded from the miRTarBase, miRDB, and TargetScan databases. The R package *limma* was used to analyze differential miRNAs and lncRNAs between high- and low-expression groups of *TAP1*, with the log fold change (logFC) > 1.5 and adjusted *p*-value < 0.05 being the thresholds to define differentially expressed miRNAs and lncRNAs. Subsequently, Cytoscape (v3.7.2) [24] was used to construct the ceRNA network by performing a correlation analysis on miRNAs that were regulated by lncRNAs and mRNAs simultaneously.

### Cell culture

C918, a human UVM cell line, was obtained from the Shanghai Cell Bank and cultured in RPMI 1640 medium supplemented with 10% fetal bovine serum, 100 units/mL penicillin, and 100 μg/mL streptomycin in a cell incubator in 5% CO_2_ and at a constant temperature of 37 °C.

### RNA interference and cell viability assay

*TAP1* siRNA sequences (Genecreate, Wuhan, China) for the designated target genes are shown in Supplementary Table S1. siRNA was transfected into C918 cells using Lipofectamine RNAiMAX reagent (Invitrogen, MA, USA) according to the manufacturer’s protocol, and the medium was changed after 24–48 h. C918 cells in log-phase growth were seeded into 96-well plates and incubated at 37 °C and in 5% CO_2_ for 24 h. Cell Counting Kit 8 (CCK8) Kit (MedChemExress, Shanghai, China) was used for CCK8 assays.

### Reverse transcriptase PCR (RT-PCR)

For RT-PCR analysis, cDNA was synthesized and quantified by real-time PCR using SYBR green dye (Vazyme, Nanjing, China). The results were normalized to the housekeeping gene *GAPDH* and expressed as 2^-ΔΔCT^ (arbitrary units) relative to the control group. The primers used are listed in Supplementary Table S2.

### Colony formation assay

C918 cells were transfected with siRNA for 48 h and seeded into 6-well plates (500 cells/well). Adherent cells were cultured in RPMI 1640 medium at 37 °C for 14 days. During this period, cells were washed every 3 days with phosphate-buffered saline (PBS), fixed with paraformaldehyde (4%) for 15 min, and then stained with crystal violet for 15 min (0.1%). Colonies of more than 50 cells were then counted. All experiments were repeated at least three times.

### Cell apoptosis and cell cycle detection

After transfection with siRNA, C918 cells were stained using apoptosis and cell cycle kits (Beyotime, Shanghai, China) according to the manufacturer’s instructions and assay signals were detected using flow cytometry.

### Cellular protein preparation and immunoblotting

Cells were harvested, washed twice in PBS, and lysed in radioimmunoprecipitation assay (RIPA) lysis buffer. The cell lysates were mixed with sodium dodecyl sulfate (SDS) sample buffer and denatured at 100 °C for 10 min. The samples were separated electrophoretically on 10–12% SDS polyacrylamide gels. Proteins were then transferred to polyvinylidene fluoride membranes and blocked with 5% skim milk for 1 h. After overnight incubation with the primary antibody (TAP1, Abclonal, China; MMP9, Cell Signaling Technology, USA; MMP2, Cell Signaling Technology, USA) at 4 °C, the membranes were washed three times with Tris-buffered saline containing Tween 20 (TBST) and incubated with horseradish peroxidase (HRP) secondary antibody for 1 h at room temperature(37 °C). Finally, membrane-bound antibodies were visualized using a protein chemiluminescent detection system.

### Statistical analysis

All data processing and analyses were performed using the R software (version 4.0.2). For comparison of two groups of continuous variables, the statistical significance of normally distributed variables was estimated using the independent Student’s *t*-test, and the differences between non-normally distributed variables were analyzed using the Mann-Whitney U test (i.e., the Wilcoxon rank-sum test). Chi-square test or Fisher’s exact test was used to compare and analyze the statistical significance between the two groups of categorical variables. Correlation coefficients between different genes were calculated using Pearson correlation analysis. The timeROC package in R [26] was used to perform survival analysis, Kaplan-Meier survival curves were used to show differences in survival, and the log-rank test was carried out to assess the significance of differences in survival time between the two groups of patients. Univariate and multivariate Cox analyses were performed to identify independent prognostic factors. Receiver operating characteristic (ROC) curves were drawn using the *pROC* package for R [27] and the area under the curve (AUC) was calculated to assess the accuracy of the risk score in estimating prognosis. All statistical *p*-values were two-sided, and *p* < 0.05 was considered statistically significant.

## Results

### Prognostic value of *TAP1* expression

**Fig. 1:**
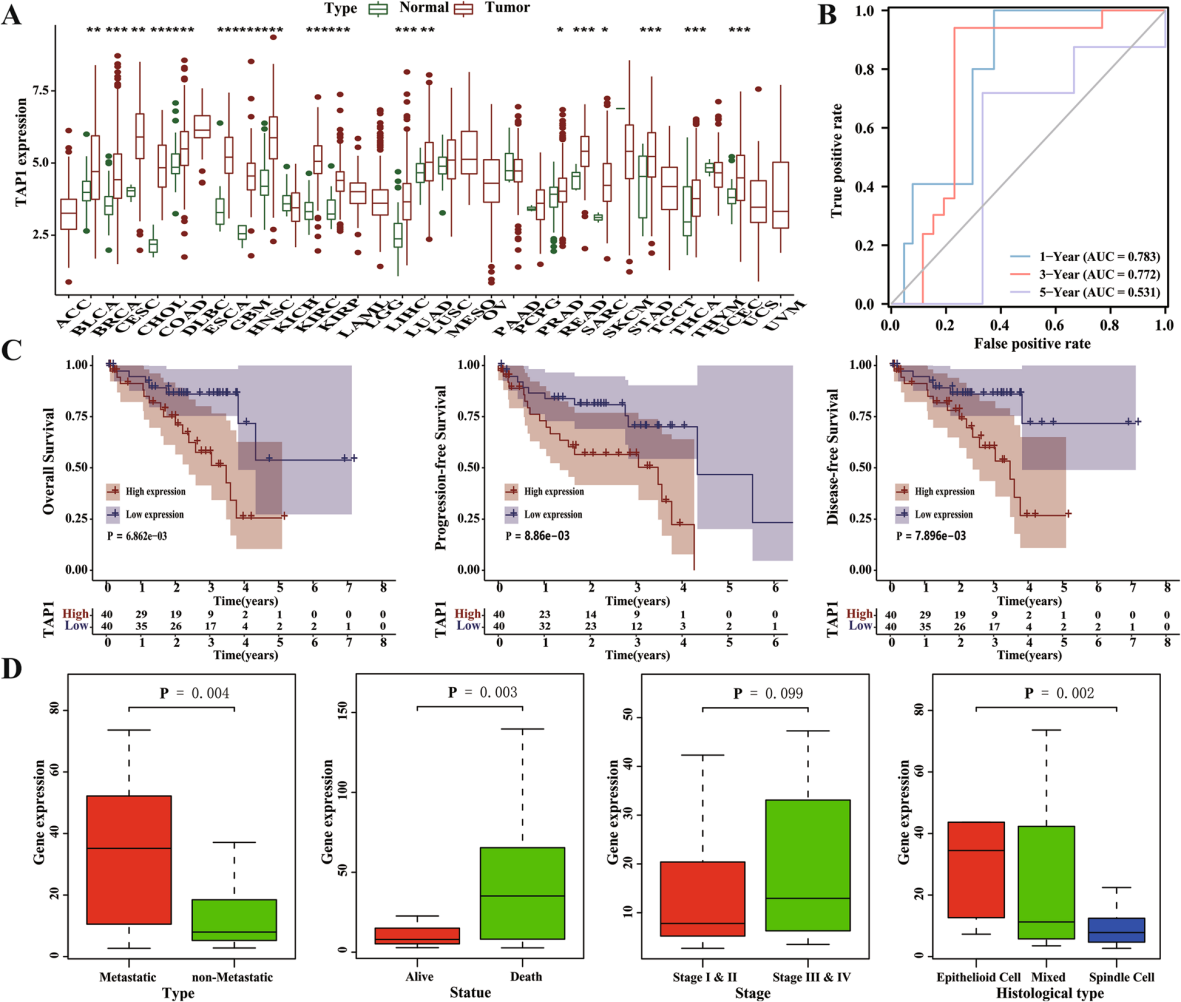
The correlation analysis of transcriptional expression of TAP1 with the prognosis and clinicopathological characteristics of UVM patients. **A** The expression of TAP1 gene in 33 tumors and adjacent tissues in the TCGA database; **B** ROC curve analysis of the predictive accuracy of TAP1 gene as a prognosis biomarker; **C** Kaplan-Meier analysis for the association between the expression of TAP1 gene with overall survival (OS), progression-free survival (PFS) and disease-free survival (DFS); **D** The correlations between TAP1 gene expression and clinicopathological features

In TCGA database, *TAP1* gene expression was significantly elevated in most tumors compared with its expression in adjacent normal tissues (Fig. 1A). ROC curve analysis showed that TAP1 could better predict the 1-, 3-, and 5-years prognosis of patients with UVM (1-, 3-, and 5-years AUC = 0.783, 0.772, and 0.531; Fig. 1B). The cut off value for ROC at each time point have been shown in supplementary Table S3. Stratified these samples into metastasis group and non-metastasis group, ROC curve analysis showed that TAP1 could better predict the 3-, and 5-years prognosis of non-metastasis patients (3-, and 5-years AUC = 0.743, and 0.754; supplementary Fig. S2A) but not in metastasis group (1-, and 3-years AUC = 0.467, and 0.257; supplementary Fig. S2B). Subsequently, we divided the patients into high- and low-expression groups according to the median value of *TAP1* expression and analyzed the association between *TAP1* expression and prognostic outcomes in OS, progression-free survival (PFS), and disease-free survival (DFS) (Fig. 1C). High expression of *TAP1* was associated with worse OS (log-rank *p* < 0.001), worse PFS (log-rank *p* < 0.001), and worse DFS (log-rank *p* < 0.001). Further validation study in GEO dataset GSE84976 showed that correlation between high expression of TAP1 and worse OS (log-rank p < 0.001) was consistent with the analysis above (supplementary Fig. S3).

### Correlation between *TAP1* gene expression and clinical features

To further analyze the correlation between *TAP1* gene expression and clinicopathological features in patients with UVM, we used the Kruskal-Wallis test and Wilcoxon rank-sum test to analyze the relationship (Fig. 1D). The results showed that high expression of the *TAP1* gene was strongly associated with a higher probability of metastasis (*p* = 0.004), higher death outcome (*p* = 0.003), and histiocytic typing (*p* = 0.002), but not with clinical stage (*p* = 0.099).

### Relationship between *TAP1* gene expression and global gene expression profiles

**Fig. 2:**
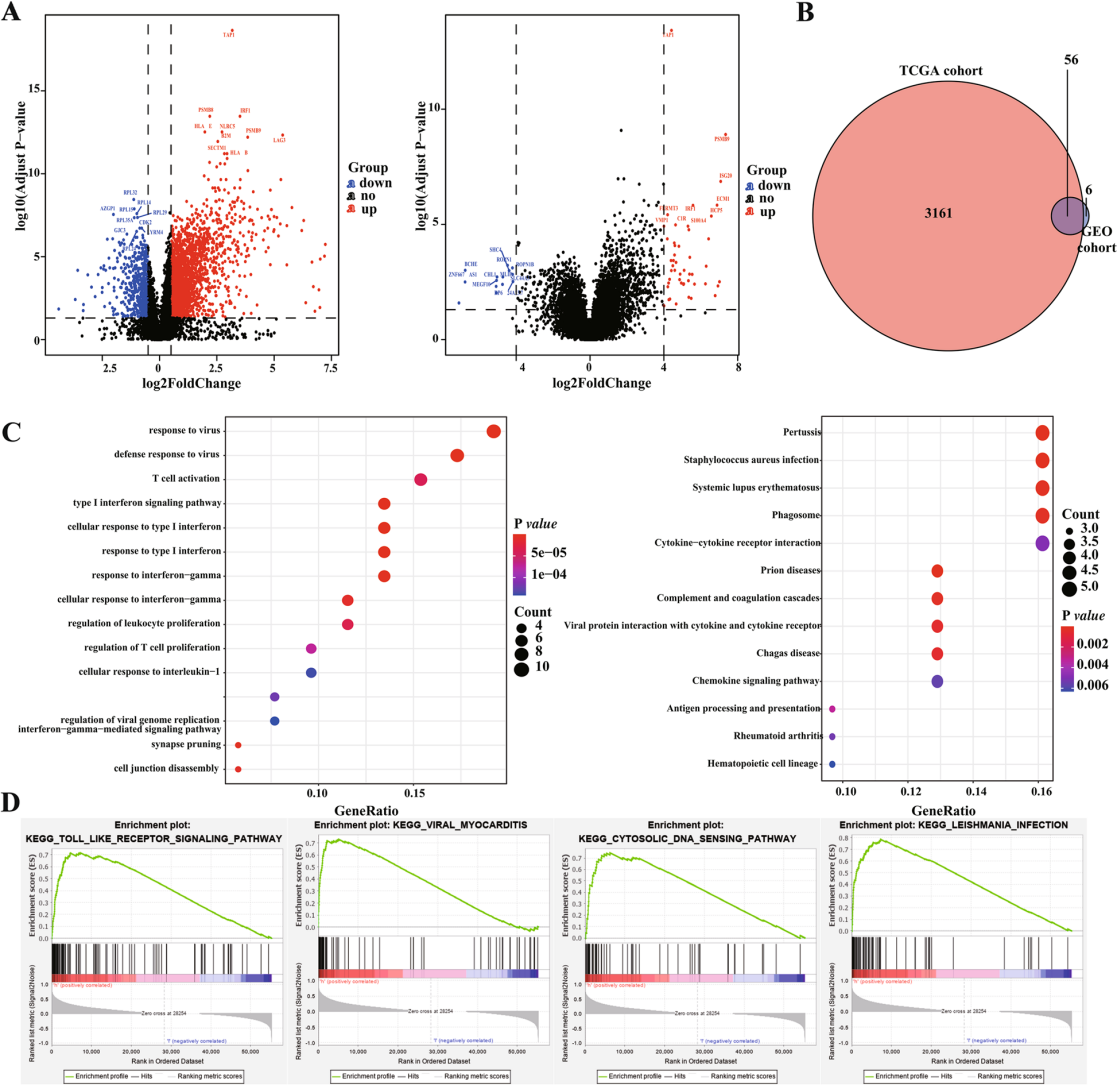
Differential gene expression and functional enrichment analysis. **A** Volcano plots show the expression of DEGs between high and low TAP1 expression groups in TCGA database and GEO database, respectively; **B** Venn plot shows the overlapping DEGs; **C** GO and KEGG analysis showed that differential genes were involved in a variety of immune-related signaling pathways; **D** GSEA analysis results showed the signal pathway that associated with TAP1 high-expressed group

To analyze the relationship between *TAP1* gene expression and the global gene expression profiles in patients with UVM, we divided the patients into high- and low-expression groups according to *TAP1* gene expression and analyzed the DEGs between the two patient groups (Fig. 2A). Using statistical thresholds of | log (fold change) | > 0.5 and FDR < 0.05, 3217 DEGs were screened in the TCGA database, whereas 62 DEGs were obtained from the GEO database. A Venn diagram analysis revealed 56 common DEGs (Fig. 2B). Subsequently, we performed a functional enrichment analysis on these common DEGs. The results of GO and KEGG functional analyses indicated that the DEGs were closely related to various immunity-related signaling pathways, such as viral infection and defense response (Fig. 2C). At the same time, GSEA showed that Toll-like receptor signaling, viral myocarditis, cytosolic and sensing pathways, Leishmania infection, and other related signaling pathways were enriched in the DEG dataset (Fig. 2D, Table 2).

**Table 2:** The results of GSEA analysis

Name	Size	Enrichment Score	NES	FDR	Leading edge
KEGG_TOLL_LIKE_RECEPTOR_SIGNALING_PATHWAY	102	0.72	1.85	0.010	tags = 49%, list = 13%, signal = 56%
KEGG_VIRAL_MYOCARDITIS	68	0.73	1.83	0.013	tags = 54%, list = 9%, signal = 60%
KEGG_CYTOSOLIC_DNA_SENSING_PATHWAY	55	0.75	1.83	0.009	tags = 47%, list = 11%, signal = 53%
KEGG_LEISHMANIA_INFECTION	70	0.79	1.81	0.009	tags = 71%, list = 13%, signal = 82%
KEGG_GALACTOSE_METABOLISM	26	0.74	1.80	0.010	tags = 65%, list = 16%, signal = 78%
KEGG_PROTEASOME	46	0.81	1.80	0.008	tags = 76%, list = 10%, signal = 85%
KEGG_APOPTOSIS	87	0.68	1.79	0.009	tags = 59%, list = 19%, signal = 72%
KEGG_RIG_I_LIKE_RECEPTOR_SIGNALING_PATHWAY	71	0.68	1.79	0.008	tags = 39%, list = 11%, signal = 44%

### PPI network and ceRNA network construction

**Fig. 3:**
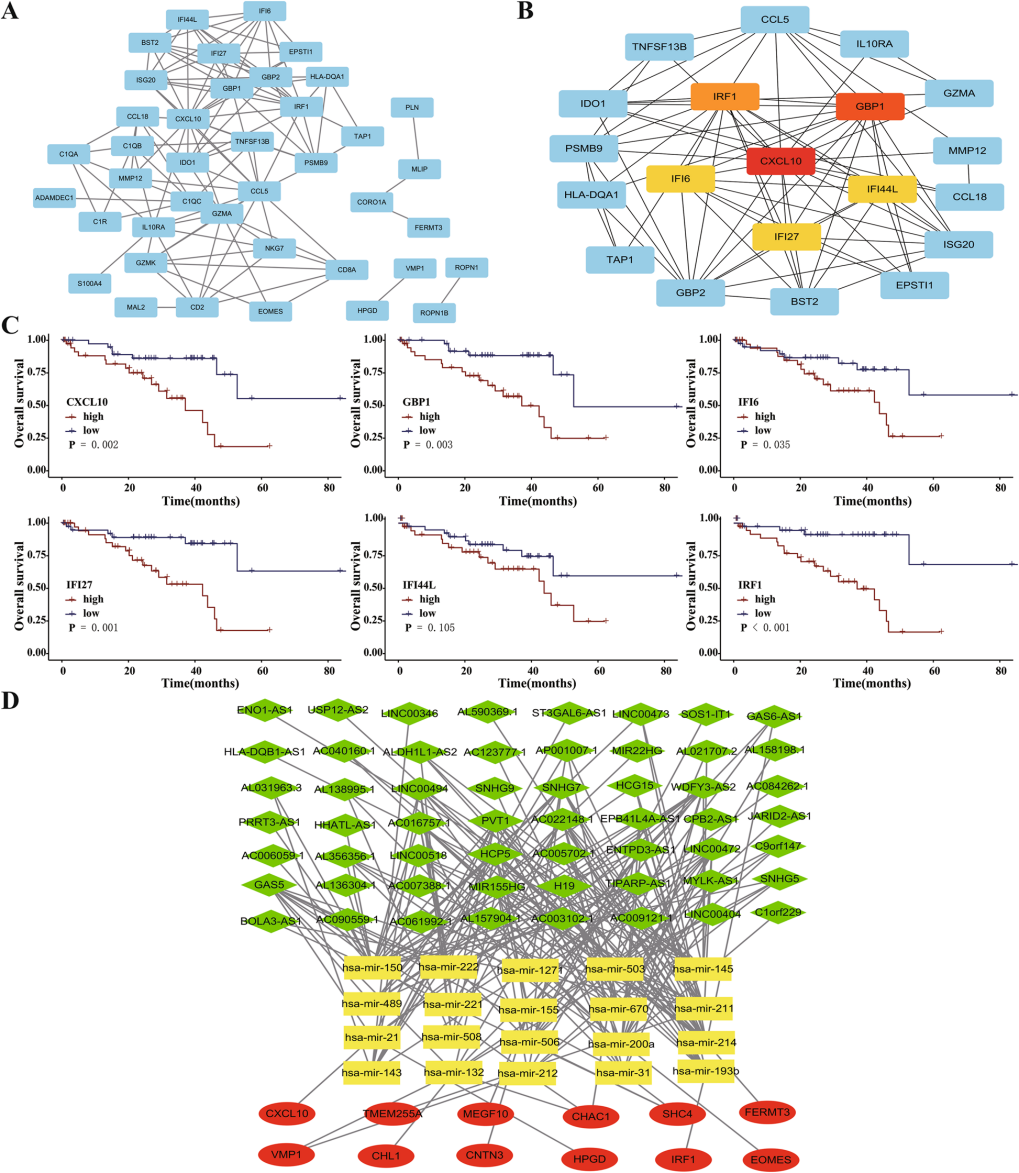
Visualization of protein-protein interaction (PPI) networks and ceRNA networks. **A** PPI network analysis for DEGs using the STRING database; **B** Hub gene selection from the entire PPI network Using the MCC algorithms; **C** Validation of the prognostic significance in hub genes based on the TCGA database; **D**The constructed ceRNA network. Oval: miRNA, diamond: lncRNA, square: mRNA

The STRING database was used to build a PPI network between the co-divergent DEGs (Fig. 3A), and the top six genes were selected from the PPI network as hub genes using the MCC algorithm with the CytoHubba plugin of Cytoscape (Fig. 3B). At the same time, we analyzed the impact of hub genes on patient prognosis (Fig. 3C). Finally, based on the differentially expressed mRNAs, miRNAs, and lncRNAs, we constructed a ceRNA network in which the three factors interacted with each other (Fig. 3D).

### Correlation analysis of *TAP1* gene expression and immune infiltration

**Fig. 4:**
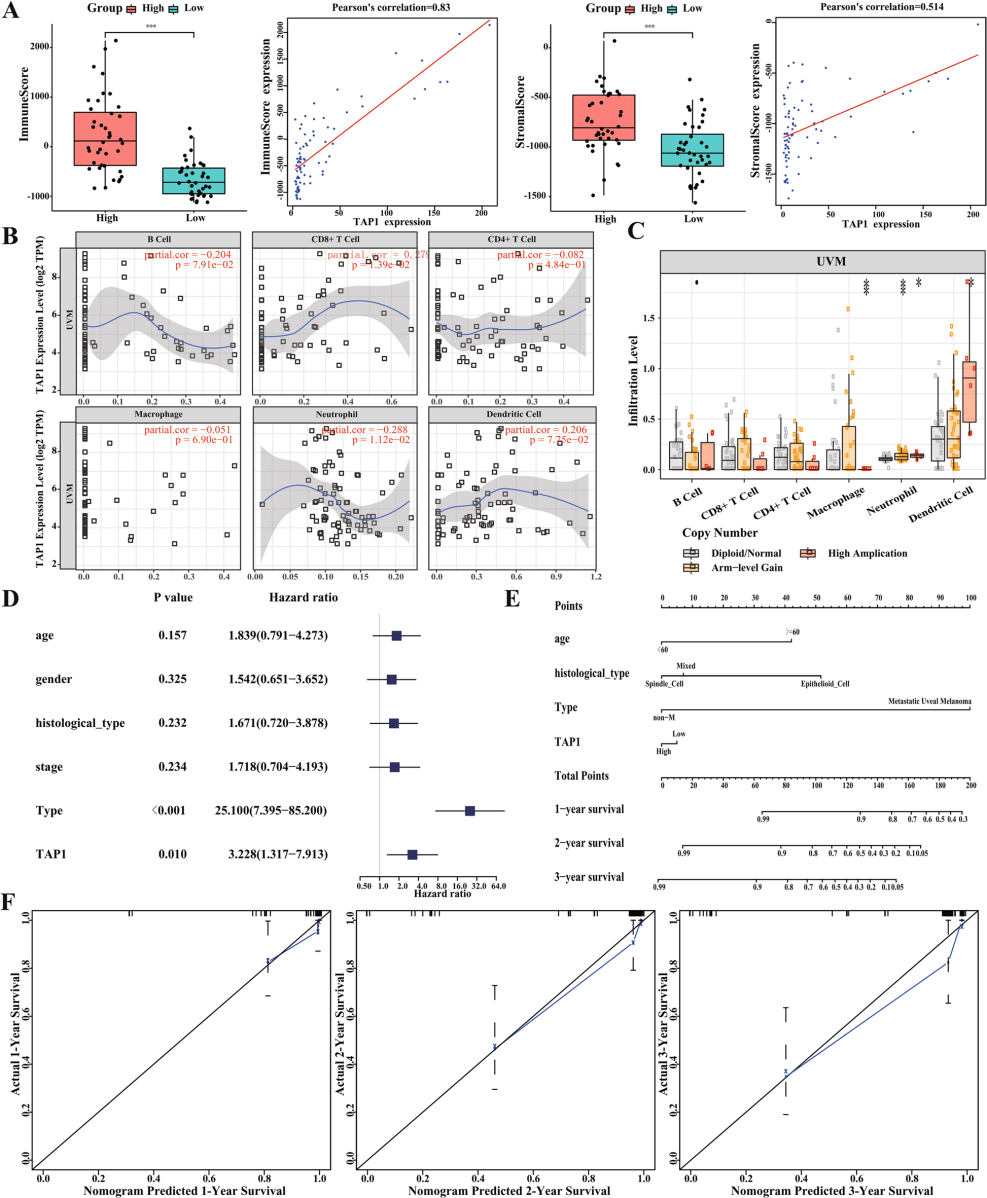
Analysis of immune infiltration and nomogram for predicting survival probabilities. **A** The high TAP1 expression group had higher immune-related scores and stroma-related scores; **B** and **C** The correlation between TAP1 expression and the level of immune cell infiltrates; **D** The univariate Cox regression analysis of the TAP1 and clinicopathologic features; **E** Integration of TAP1 and clinical characteristics to construct Nomogram for predicting OS of UVM patients; **F** Calibration plots for the nomograms showed that the constructed nomogram had higher prognostic efficiency at 1, 2, and 3 years; The X-axis represents the nomogram-predicted survival, and the actual survival is plotted on the Y-axis

To further analyze the function of *TAP1*, we performed immune microenvironment analysis. We assessed the correlation between the level of *TAP1* gene expression, immune characteristics, and immune cell subsets in patients with UVM in the TCGA database. Tumor tissues not only contain tumor cells but also a variety of different cell types, such as stromal cells, fibroblasts, and immune cells, constituting the tumor microenvironment. Stromal cells play an important role in the development, metastasis, and drug resistance of tumors. The results showed that the *TAP1* high-expression group had higher immune-related and stroma-related scores than the low-expression group, and there was a significant positive correlation between the expression level, immune-related score, and stroma-related score (Fig. 4A). Subsequently, the TIMER database was used to analyze the effects of *TAP1* expression and mutation levels on immune cell infiltration in patients with UVM. In these patients, the expression of TAP1 was positively correlated with CD8^+^ T cells and DCs, but was negatively correlated with B cells and neutrophils (Fig. 4B). The infiltration levels of multiple immune cells were also significantly different in tissues with different mutant forms of TAP1 (Fig. 4C). These results confirmed the association of TAP1 with stromal and immune cells in the UVM immune microenvironment.

### Construction of a nomogram to predict OS of patients with UVM

We also performed univariate and multivariate Cox regression analyses for OS prognosis (Fig. 4D, Table 3), and the results showed that high and low expression of *TAP1* were closely related to OS (HR = 3.228 (1.317–7.913), *p* = 0.010; Table 3), but TAP1 was not an independent risk factor for prognosis. We incorporated the expression of the *TAP1* gene and clinicopathological features into the model and constructed a nomogram to predict OS in patients with UVM (Fig. 4E). The C-index was used to calculate the discriminatory power of the nomogram, which showed a high degree of discrimination (0.903 [0.842–0.964]). Moreover, we tested the predictive efficiency of the model using calibration plots. There was a strong agreement between the estimated OS and the actual observed OS values of the patients at 1, 2, and 3 years. The results suggested that the constructed OS prognostic model could accurately predict the OS of patients at 1, 2, and 3 years (Fig. 4F).

**Table 3:** The univariate and multivariate Cox regression analysis for OS in TCGA database based on the expression of *TAP1*

Variables	Univariate Cox analysis		Multivariate Cox analysis	
	HR (95% CI)	*P* value	HR (95% CI)	*P* value
Age (≥60 vs. < 60)	1.83(0.79–4.27)	0.157	10.33(2.20–48.61)	0.003^**^
Gender (male vs. female)	1.54(0.65–3.65)	0.325	0.95(0.29–3.11)	0.938
Histological type (Mixed vs. Others)	1.67(0.72–3.88)	0.232	0.33(0.11–0.98)	0.045^*^
Stage (III + IV vs. I + II)	1.72(0.70–4.19)	0.234	3.82(1.13–12.91)	0.031^*^
Type (metastatic vs. non- metastatic)	25.10(7.39–85.20	< 0.001^***^	75.28(15.92–355.91)	< 0.001^***^
*TAP1* (high vs. low)	3.23(1.32–7.91)	0.010^*^	1.43(0.53–3.87)	0.484

### Detection of proliferation, invasion, and metastasis in vitro

**Fig. 5:**
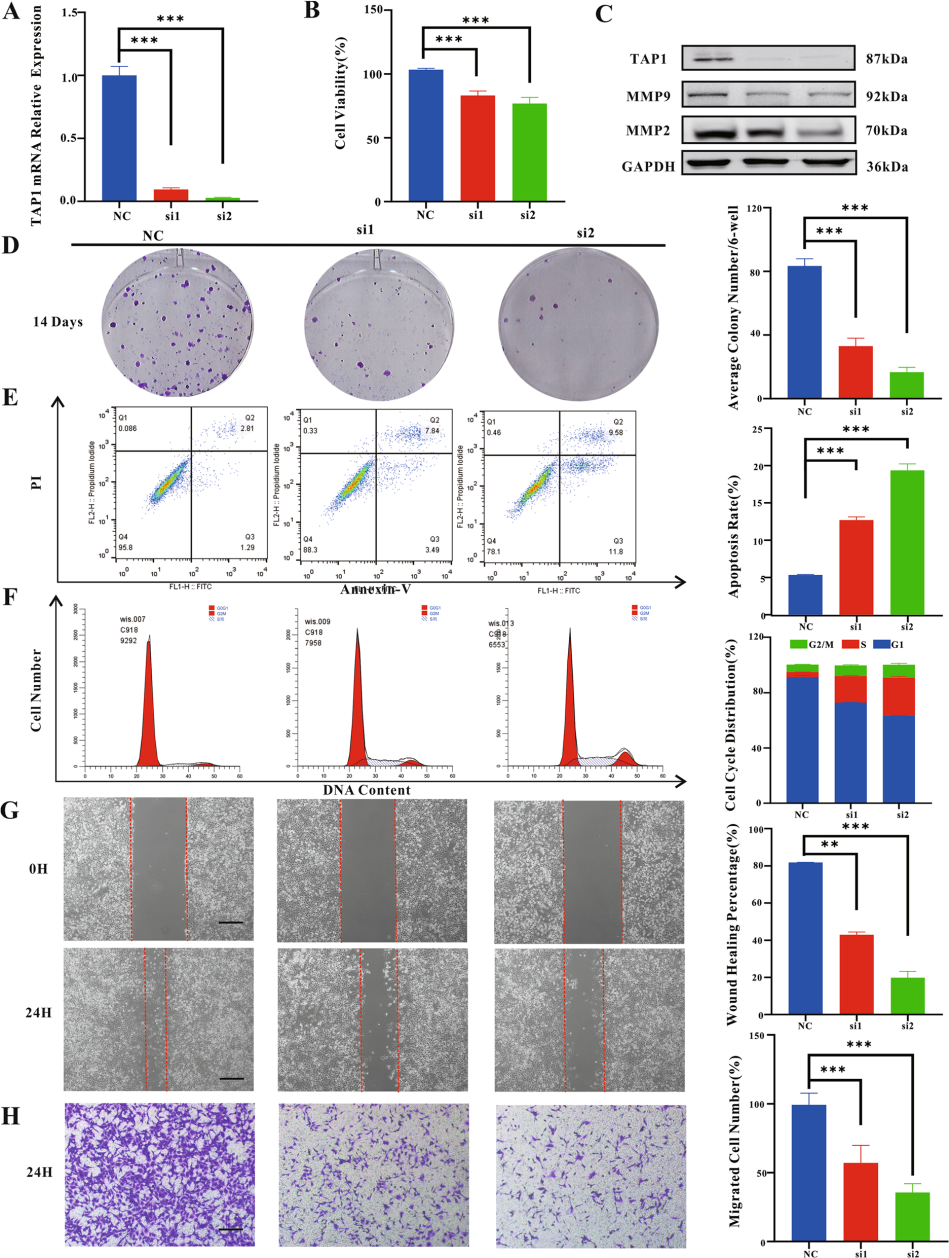
Effects of silencing TAP1 expression on UVM cells In vitro. **A** PCR showed that siRNA successfully silenced the mRNA expression of TAP1; **B** CCK8 experiment showed that silencing TAP1 significantly inhibited the proliferation activity of C918; **C** WB experiment showed that siRNA successfully inhibited the protein expression of TAP1, and the protein expression of MMP9 and MMP2 also decreased; **D** Colony formation assay showed that silencing TAP1 significantly inhibited the growth activity of C918; **E** Apoptosis flow cytometry showed that the number of tumor cells apoptotic was significantly increased; **F** The cell cycle assay showed that tumor cell cycle was blocked in S phase; **G-H** Scratch and transwell experiments showed that the tumor cells in the siRNA group had significantly reduced ability to metastasize and migrate. The scale bar represents 50 μm in all the pictures

To determine the effect of TAP1 on the proliferative activity and metastatic migration ability of tumor cells in UVM, we successfully knocked down *TAP1* expression in C918 cells using *TAP1* siRNA (Fig. 5A and C). CCK8 and colony formation assays showed a significant decrease in tumor cell proliferation activity and cell colony formation in both the silenced groups (Fig. 5B and D). To explain the mechanism by which silenced *TAP1* reduces C918 cell viability, we assessed apoptosis caused by *TAP1* inhibition. After annexin V/propidium iodide staining, the number of annexin V-positive cells increased significantly after *TAP1* knockdown, indicating that silencing *TAP1* could induce tumor cell apoptosis (Fig. 5E). S-phase arrest was subsequently observed in the cell cycle experiments (Fig. 5F). We further examined the effects of TAP1 on the metastatic and migratory abilities of C918 cells. At the 24th hour after the scratch test, the scratches in the control group were mostly healed, while the healing rate of the *TAP1* low-expression group was only 20–40% (Fig. 5G). Silencing of *TAP1* reduced the number of cells migrating through the membrane to the bottom of the well (Fig. 5H). Western blot experiments further showed that the expression levels of the tumor metastasis markers, matrix metallopeptidase 9 (MMP9) and MMP2, were decreased significantly in C918 cells after *TAP1* silencing (Fig. 5C). Based on these results, *TAP1* expression appeared to correlate significantly with in vitro proliferation, invasion, and metastatic ability of C918 cells.

## Discussion

UVM is a highly malignant form of melanoma and a rare subset of cancers resistant to immune checkpoint blockade [28, 29]. Immunotherapy has made substantial progress in treating cutaneous melanoma but it rarely produces similar treatment responses when treating UVM [3]. Thus, finding new immunotherapeutic targets to improve UVM treatment is crucial. The TAP family, which comprises TAP1 and TAP2, plays a vital role in the transportation of antigenic peptides. They are responsible for peptide delivery between the cytoplasm and the lumen of the endoplasmic reticulum, as well as the loading of MHC I molecules [9], making endogenous peptides available for the recognition of CD8+ cytotoxic T cells [13, 14]. Thus, the TAP family of transporters serves to maintain the proper functioning of the immune system. In this study, the prognostic value and immune-related characteristics of TAP1 were explored in vitro to provide insights into novel prognostic biomarkers and therapeutic targets for UVM.

TAP1 has been reported to be closely associated with a variety of tumors and may play a role in facilitating immune evasion by interfering with peptide delivery [10]. Agnes Ling et al. revealed a strong correlation between down-regulation of TAP1 and poor prognosis in stage I-II CRC patients [30]. Anika Tabassum et al. found that prognostic analysis on the OV showed a positive correlation for TAP1 expression, while it is negative on BRCA, LIHC, and LUAD [11]. In patients with ovarian cancer, TAP1 protein abundance was negatively correlated with survival time and significantly correlated with immune infiltration and poor prognosis [12].Therefore, there is no consistent relationship between *TAP1* expression and patient prognosis in different cancer types.

In this study, through the analysis of data from TCGA and GEO databases, *TAP1* expression was found to be significantly elevated in UVM, and the clinical value of *TAP1* mRNA expression was explored in different clinical variables such as histological type. In addition, by constructing a prognostic analysis model for OS, *TAP1* expression could accurately predict the 1-, 3-, and 5-years survival of patients. Since metastasis status is the most influential predictor in nomogram, we investigated TAP1 as and prognosis predictor after excluding all metastasis sample. The ROC curve analysis indicated that the TAP1 expression can predict the 3-, and 5-years prognosis of non-metastasis patients accurately. These results suggest that TAP1 may act as a prognostic marker for UVM.

Analysis of *TAP1* expression and global gene expression profiles in patients with UVM showed that DEGs were closely related to various immunity-related signaling pathways, such as viral infection and defense response. According to the GSEA analysis, multiple immunity-related signaling pathways, such as primary immunodeficiency, DNA replication, and the cell cycle of the Toll-like receptor signaling pathway, were enriched alongside elevated *TAP1* expression. Therefore, TAP1 protein might play a role in the regulation of UVM immunity.

TAP1 also plays a role in tumor development and treatment resistance, mainly by affecting tumor immune infiltration. Loss of *TAP1* expression in cutaneous melanoma is associated with an increase in regulatory T (Treg) cells and neutrophils in cancer, which might alter the immune microenvironment and be involved in reversing resistance to PD1 therapy [13, 14]. The level of immune cell infiltration correlated significantly with *TAP1* expression levels. Analysis of the TIMER database showed that TAP1 is also associated with the infiltration of various immune cells. The higher the transcriptional level of the *TAP1* gene, the higher the degree of infiltration of CD8^+^ T cells and DCs. However, *TAP1* expression was negatively correlated with the number of B cells and neutrophils. The difference in the infiltration of immune cells might be responsible for the correlation between *TAP1* expression in different tumors and patient survival, thus requiring further investigation. Overall, these results suggest that the effect of *TAP1* gene expression on the tumor microenvironment may have an important role in the occurrence, development, metastasis, and immune response of UVM.

To analyze the function of TAP1, we also identified 100 genes that interact directly with TAP1 from the PPI network database and jointly constructed an interaction network module dominated by TAP1-interacting proteins. CXCL10 is involved in the regulation of multiple proinflammatory cytokines by stimulating the activation and migration of immune cells to the site of infection [31]. The related pathways include the interaction between immune cells and microRNAs in the tumor microenvironment and the CCR5 pathway in macrophages [32]. *GBP1* and *IFI27* are also related regulatory genes involved in immunomodulation and interferon responses [33, 34]. Among these, IFI6 and IFI44L are associated with the formation and growth of cutaneous melanoma [35, 36]. The interaction network diagram showed that several oncogenes and immunity-related genes interact directly with *TAP1*. The association between these oncogenes, immunity-related genes, and *TAP1* suggests that part of the poor prognosis in UVM may be related to multiple regulations of tumor invasion and the tumor microenvironment.

Subsequently, we constructed a ceRNA network via mRNA–miRNA–lncRNA sequential patterns. In this network, we found that many genes were directly associated with various cancers. *SHC4* promotes tumor proliferation and metastasis in melanoma and hepatocellular carcinoma [37, 38], whereas *MEGF10* modulates the cellular aggressiveness of UVM [39]. *CHAC1*, a ferroptosis-related gene, is correlated with breast cancer [40]. Furthermore, *EOMES* is involved in the differentiation of CD8+ T-cells during immune response [41]. The findings from the ceRNA network further proved that TAP1 is essential for UVM initiation, progression, metastasis, and the tumor microenvironment.

We further investigated the role of TAP1 in the proliferation of C918 cells in vitro. After silencing *TAP1* expression successfully with siRNA, we found that the knockdown of *TAP1* significantly inhibited the growth activity of C918 cells. Flow cytometry revealed increased apoptosis and cell cycle arrest in C918 cells with low *TAP1* expression. Transwell assays and scratch experiments demonstrated that TAP1 is significantly associated with the metastatic and migratory abilities of C918 cells. Further detection of related metastasis indicators revealed that the expression levels of the metastasis-related indicators MMP9 and MMP2 also decreased upon *TAP1* silencing.

Nevertheless, this study has several limitations. First, TAP1 was identified as a prognostic biomarker only at the mRNA level; further studies should be conducted to verify the potential of TAP1 as a biomarker. Second, subsequent studies are needed to confirm the molecular mechanisms of the TAP1-associated pathways. Third, although studies using cell lines have a certain reference value for the development of drugs targeting TAP1, the transfection experiments in this study were only performed on the C918 cell line. Thus, the mechanism of action and animal study are required to further verify the exact role of TAP1 in UVM in vitro status. Finally, the association between TAP1 and tumor growth and the immune microenvironment of UVM as well as its mechanism of action would require further investigation.

## Conclusions

In summary, our results suggest that *TAP1* expression is increased in UVM and is positively correlated with poor prognosis and immune cell infiltration. Furthermore, we found that TAP1 affects UVM development in different ways through cell proliferation and metastatic migration. Consequently, our findings suggest that *TAP1* may act as a prognostic biomarker and potential new therapeutic target for UVM.

## Supplementary Information

**Additional file 1: Supplementary Fig. S1.** Original data of Western Blot. **Supplementary Fig. S2.** ROC curve analysis. (**A**) ROC curve analysis of the predictive accuracy of *TAP1* gene as a prognosis biomarker in non-metastasis group; (**B**) ROC curve analysis of the predictive accuracy of *TAP1* gene as a prognosis biomarker in metastasis group. **Supplementary Fig. S3.** Overall survival analysis for GSE84976. Kaplan-Meier analysis for the association between the expression of TAP1 gene with overall survival (OS) in GSE84976 dataset. **Supplementary Table S1:** siRNA sequence. **Supplementary Table S2:** Primer sequence. **Supplementary Table S3:** Cut off value of AUC.

## Data Availability

The datasets generated and analysed during the current study are available in GEO dataset (https://www.ncbi.nlm.nih.gov/geo/) and TCGA dataset (https://portal.gdc.cancer.gov/).

## References

[1] Fallico M, Raciti G, Longo A, Reibaldi M, Bonfiglio V, Russo A (2021). Current molecular and clinical insights into uveal melanoma (review). Int J Oncol..

[2] Jager MJ, Shields CL, Cebulla CM, Abdel-Rahman MH, Grossniklaus HE, Stern MH, Carvajal RD, Belfort RN, Jia R, Shields JA (2020). Uveal melanoma. Nat Rev Dis Primers.

[3] Fu Y, Xiao W (2022). Recent Advances and Challenges in Uveal Melanoma Immunotherapy. Cancers (Basel).

[4] Levidou G, Gajdzis P, Cassoux N, Donizy P, Masaoutis C, Gajdzis M, Gardrat S, Pergaris A, Danas E, Klijanienko J (2021). Histone deacetylase (HDAC)-1, −2, −4, and −6 in uveal melanomas: associations with Clinicopathological parameters and Patients' survival. Cancers.

[5] Kashyap S, Jha J, Singh MK, Singh L, Sen S, Kaur J, Bajaj MS, Pushker N (2020). DNA damage response proteins and its role in tumor progression of uveal melanoma with patient outcome. Clinical & translational oncology : official publication of the Federation of Spanish Oncology Societies and of the National Cancer Institute of Mexico.

[6] Singh MK, Singh L, Pushker N, Saini N, Meel R, Chosdol K, Bakhshi S, Sen S, Venkatesh P, Chawla B (2019). Identification of canonical NFκB (C-NFκB) pathway in uveal melanoma and their relation with patient outcome. Clin Exp Metastasis.

[7] Gallenga CE, Franco E, Adamo GG, Violanti SS, Tassinari P, Tognon M, Perri P (2022). Genetic basis and molecular mechanisms of uveal melanoma metastasis: a focus on prognosis. Front Oncol.

[8] Lane AM, Kim IK, Gragoudas ES (2018). Survival rates in patients after treatment for metastasis from uveal melanoma. JAMA ophthalmology.

[9] Leone P, Shin EC, Perosa F, Vacca A, Dammacco F, Racanelli V (2013). MHC class I antigen processing and presenting machinery: organization, function, and defects in tumor cells. J Natl Cancer Inst.

[10] Meng J, Li W, Zhang M, Hao Z, Fan S, Zhang L, Liang C (2018). An update meta-analysis and systematic review of TAP polymorphisms as potential biomarkers for judging cancer risk. Pathol Res Pract.

[11] Tabassum A, Samdani MN, Dhali TC, Alam R, Ahammad F, Samad A, Karpiński TM (2021). Transporter associated with antigen processing 1 (TAP1) expression and prognostic analysis in breast, lung, liver, and ovarian cancer. J Mol Med (Berl).

[12] Li X, Zeng S, Ding Y, Nie Y, Yang M (2021). Comprehensive analysis of the potential immune-related biomarker transporter associated with antigen processing 1 that inhibits metastasis and invasion of ovarian Cancer cells. Front Mol Biosci.

[13] Wang Y, Yan K, Lin J, Liu Y, Wang J, Li X, Li X, Hua Z, Zheng Z, Shi J (2020). CD8+ T cell co-expressed genes correlate with clinical phenotype and microenvironments of urothelial Cancer. Front Oncol.

[14] Zhang X, Sabio E, Krishna C, Ma X, Wang J, Jiang H, Havel JJ (2021). Qa-1(b) Modulates Resistance to Anti-PD-1 Immune Checkpoint Blockade in Tumors with Defects in Antigen Processing. Mol Cancer Res.

[15] Laurent C, Valet F, Planque N, Silveri L, Maacha S, Anezo O, Hupe P, Plancher C, Reyes C, Albaud B (2011). High PTP4A3 phosphatase expression correlates with metastatic risk in uveal melanoma patients. Cancer Res.

[16] van Essen TH, van Pelt SI, Bronkhorst IH, Versluis M, Némati F, Laurent C, Luyten GP, van Hall T, van den Elsen PJ, van der Velden PA (2016). Upregulation of HLA expression in primary uveal melanoma by infiltrating leukocytes. PLoS One.

[17] Ritchie ME, Phipson B, Wu D, Hu Y, Law CW, Shi W, Smyth GK (2015). Limma powers differential expression analyses for RNA-sequencing and microarray studies. Nucleic Acids Res.

[18] Chen H, Boutros PC (2011). VennDiagram: a package for the generation of highly-customizable Venn and Euler diagrams in R. BMC bioinformatics.

[19] Kanehisa M, Sato Y, Furumichi M, Morishima K, Tanabe M (2019). New approach for understanding genome variations in KEGG. Nucleic Acids Res.

[20] Yu G, Wang LG, Han Y, He QY (2012). clusterProfiler: an R package for comparing biological themes among gene clusters. Omics : a journal of integrative biology.

[21] Subramanian A, Tamayo P, Mootha VK, Mukherjee S, Ebert BL, Gillette MA, Paulovich A, Pomeroy SL, Golub TR, Lander ES (2005). Gene set enrichment analysis: a knowledge-based approach for interpreting genome-wide expression profiles. Proc Natl Acad Sci U S A.

[22] Li T, Fan J, Wang B, Traugh N, Chen Q, Liu JS, Li B, Liu XS (2017). TIMER: a web server for comprehensive analysis of tumor-infiltrating immune cells. Cancer Res.

[23] Yoshihara K, Shahmoradgoli M, Martínez E, Vegesna R, Kim H, Torres-Garcia W, Treviño V, Shen H, Laird PW, Levine DA (2013). Inferring tumour purity and stromal and immune cell admixture from expression data. Nat Commun.

[24] Shannon P, Markiel A, Ozier O, Baliga NS, Wang JT, Ramage D, Amin N, Schwikowski B, Ideker T (2003). Cytoscape: a software environment for integrated models of biomolecular interaction networks. Genome Res.

[25] Chin CH, Chen SH, Wu HH, Ho CW, Ko MT (2014). Lin CY: cytoHubba: identifying hub objects and sub-networks from complex interactome. BMC Syst Biol.

[26] Liu J, Lichtenberg T, Hoadley KA, Poisson LM, Lazar AJ, Cherniack AD, Kovatich AJ, Benz CC, Levine DA, Lee AV (2018). An integrated TCGA Pan-Cancer clinical data resource to drive high-quality survival outcome analytics. Cell.

[27] Robin X, Turck N, Hainard A, Tiberti N, Lisacek F, Sanchez JC, Müller M (2011). pROC: an open-source package for R and S+ to analyze and compare ROC curves. BMC bioinformatics.

[28] Wessely A, Steeb T, Erdmann M, Heinzerling L, Vera J, Schlaak M (2020). The role of immune checkpoint blockade in uveal melanoma. Int J Mol Sci..

[29] Shain AH, Bagger MM, Yu R, Chang D (2019). The genetic evolution of metastatic uveal melanoma. Nat Genet.

[30] Ling A, Löfgren-Burström A, Larsson P, Li X, Wikberg ML, Öberg Å, Stenling R, Edin S, Palmqvist R (2017). TAP1 down-regulation elicits immune escape and poor prognosis in colorectal cancer. Oncoimmunology.

[31] Reschke R, Gajewski TF (2022). CXCL9 and CXCL10 bring the heat to tumors. Sci Immunol.

[32] Halama N, Zoernig I, Berthel A, Kahlert C, Klupp F, Suarez-Carmona M, Suetterlin T, Brand K, Krauss J, Lasitschka F (2016). Tumoral immune cell exploitation in colorectal Cancer metastases can be targeted effectively by anti-CCR5 therapy in Cancer patients. Cancer Cell.

[33] Fisch D, Bando H, Clough B, Hornung V, Yamamoto M, Shenoy AR, Frickel EM (2019). Human GBP1 is a microbe-specific gatekeeper of macrophage apoptosis and pyroptosis. EMBO J.

[34] Huang H, Lv J, Huang Y, Mo Z, Xu H, Huang Y, Yang L, Wu Z, Li H, Qin Y (2022). IFI27 is a potential therapeutic target for HIV infection. Ann Med.

[35] Gupta R, Forloni M, Bisserier M, Dogra SK, Yang Q, Wajapeyee N (2016). Interferon alpha-inducible protein 6 regulates NRASQ61K-induced melanomagenesis and growth. Elife.

[36] Puig-Butille JA, Escámez MJ, Garcia-Garcia F, Tell-Marti G, Fabra À, Martínez-Santamaría L, Badenas C, Aguilera P, Pevida M, Dopazo J (2014). Capturing the biological impact of CDKN2A and MC1R genes as an early predisposing event in melanoma and non melanoma skin cancer. Oncotarget.

[37] Fagiani E, Giardina G, Luzi L, Cesaroni M, Quarto M, Capra M, Germano G, Bono M, Capillo M, Pelicci P (2007). RaLP, a new member of the Src homology and collagen family, regulates cell migration and tumor growth of metastatic melanomas. Cancer Res.

[38] Zhang X, Zhang H, Liao Z, Zhang J, Liang H, Wang W, Yu J, Dong K (2022). SHC4 promotes tumor proliferation and metastasis by activating STAT3 signaling in hepatocellular carcinoma. Cancer Cell Int.

[39] Yang H, Cai MY, Rong H, Ma LR, Xu YL (2021). ZNF667-AS1, a positively regulating MEGF10, inhibits the progression of uveal melanoma by modulating cellular aggressiveness. J Biochem Mol Toxicol.

[40] Mehta V, Suman P, Chander H (2022). High levels of unfolded protein response component CHAC1 associates with cancer progression signatures in malignant breast cancer tissues. Clin Transl Oncol.

[41] Koetzier SC, van Langelaar J, Melief MJ, Wierenga-Wolf AF, Corsten CEA (2022). Distinct Effector Programs of Brain-Homing CD8(+) T Cells in Multiple Sclerosis. Cells.

