# Supplementary material for: TAP1, a potential immune-related prognosis biomarker with functional significance in uveal melanoma
Source: BMC Cancer. 2023 Feb 11;23:146. doi: 10.1186/s12885-023-10527-9 (PMC9921415; doi:10.1186/s12885-023-10527-9)
Supplement: Supplementary file 1 — Additional file 1: Supplementary Fig. S1. Original data of Western Blot. Supplementary Fig. S2. ROC curve analysis. (A) ROC curve analysis of the predictive accuracy of TAP1 gene as a prognosis biomarker in non-metastasis group; (B) ROC curve analysis of the predictive accuracy of TAP1 gene as a prognosis biomarker in metastasis group. Supplementary Fig. S3. Overall survival analysis for GSE84976. Kaplan-Meier analysis for the association between the expression of TAP1 gene with overall survival (OS) in GSE84976 dataset. Supplementary Table S1: siRNA sequence. Supplementary Table S2: Primer sequence. Supplementary Table S3: Cut off value of AUC. [file 12885_2023_10527_MOESM1_ESM.pdf]

Supplementary Information

TAP1, a Potential Immune-Related Prognosis Biomarker with Functional Significance in Uveal Melanoma

Ru Zhu[1, †], Yu-Ting Chen[2, †], Bo-Wen Wang [1], Ya-Yan You[1], Xing-Hua Wang[1], Hua-Tao Xie[1], Fa-Gang Jiang [1, *], Ming-Chang Zhang [1, *]

[1] Department of Ophthalmology, Union Hospital, Tongji Medical College, Huazhong University of Science and Technology, Wuhan 430022, China

[2] Cancer Center, Union Hospital, Tongji Medical College, Huazhong University of Science and Technology, Wuhan 430022, China

[†]These authors contributed equally

[*]Correspondence:

Ming-Chang Zhang; Fa-Gang Jiang

Department of Ophthalmology, Union Hospital, Tongji Medical College, Huazhong University of Science and Technology, No. 1277 Jiefang Avenue, Wuhan, Hubei Province, 430022, People's Republic of China Tel: (86)27-88661897, Fax: (86)27-88665610

Email: mingchangzhang@hotmail.com; fgjiang@hotmail.com

Keywords: TAP1，Uveal melanoma, prognostic biomarkers, immunomodulation, metastasis

Materials and methods

Data download

The gene expression data (FPKM values) from the RNA sequencing of 80 patients with UVM were downloaded from the official website of The Cancer Genome Atlas (TCGA) Genomic Data Commons (GDC) (https://portal.gdc.cancer.gov/) and divided into mRNA and long noncoding RNA (lncRNA) expression. The clinicopathological features and prognosis of the individual patients with UVM, such as sex, age, and stage, were downloaded from the University of California, Santa Cruz (UCSC) website (http://xena.ucsc.edu/). The specific clinical information on these patients is presented in Table 1. Moreover, the gene expression data of GSE221381 [1] and clinicopathological features of the patients were downloaded from the Gene Expression Omnibus (GEO) database as the validation dataset. The data originated from *Homo sapiens* samples and were generated on the GPL570[HG-U133_Plus_2] Affymetrix Human Genome U133 Plus 2.0 Array platform, including 63 UVM tissue samples. We also downloaded the gene expression data of GSE84976 [2] and clinicopathological features of the patients GEO database. The data originated from Homo sapiens samples and were generated on the GPL10558 Illumina HumanHT-12 V4.0 expression beadchip Array platform, including 28 UVM tissue samples. as a validation dataset.

Construction and validation of clinical prediction models

To further evaluate the influence of *TAP1* gene expression combined with clinicopathological features on patient prognosis, univariate and multivariate Cox

analysis risk scores were used in combination with clinicopathological features to independently predict overall survival (OS). Samples were stratified into metastasis group and non-metastasis group， and then multivariate Cox analysis risk scores were used to independently predict overall survival (OS), separately.

Cell culture

C918, a human UVM cell line, was obtained from the Shanghai Cell Bank and cultured in RPMI 1640 medium supplemented with 10% fetal bovine serum, 100 units/mL penicillin, and 100 μg/mL streptomycin in a cell incubator in 5% CO_2 and at a constant temperature of 37 °C.

RNA interference

TAP1 siRNA sequences (Genecreate, Wuhan, China) for the designated target genes are shown in Supplementary Table S1. siRNA was transfected into C918 cells using Lipofectamine RNAiMAX reagent (Invitrogen, MA, USA) according to the manufacturer's protocol, and the medium was changed after 24–48 h.

Cellular protein preparation and immunoblotting

Cells were harvested, washed twice in PBS, and lysed in radioimmunoprecipitation assay (RIPA) lysis buffer. The cell lysates were mixed with sodium dodecyl sulfate (SDS) sample buffer and denatured at 100 °C for 10 min. The samples were separated electrophoretically on 10–12% SDS polyacrylamide gels. Proteins were then transferred to polyvinylidene fluoride membranes and blocked with 5% skim milk for

1 h. After overnight incubation with the primary antibody (TAP1, Abclonal, China; MMP9, Cell Signaling Technology, USA; MMP2, Cell Signaling Technology, USA) at 4 °C, the membranes were washed three times with Tris-buffered saline containing Tween 20 (TBST) and incubated with horseradish peroxidase (HRP) secondary antibody for 1 h at room temperature(37 °C). Finally, membrane-bound antibodies were visualized using a protein chemiluminescent detection system.

Statistical analysis

All data processing and analyses were performed using the R software (version 4.0.2). For comparison of two groups of continuous variables, the statistical significance of normally distributed variables was estimated using the independent Student's *t*-test, and the differences between non-normally distributed variables were analyzed using the Mann-Whitney U test (i.e., the Wilcoxon rank-sum test). Chi-square test or Fisher's exact test was used to compare and analyze the statistical significance between the two groups of categorical variables. Correlation coefficients between different genes were calculated using Pearson correlation analysis. The timeROC package in R [3] was used to perform survival analysis, Kaplan-Meier survival curves were used to show differences in survival, and the log-rank test was carried out to assess the significance of differences in survival time between the two groups of patients. Univariate and multivariate Cox analyses were performed to identify independent prognostic factors. Receiver operating characteristic (ROC) curves were drawn using the *pROC* package for R [4] and the area under the curve (AUC) was

calculated to assess the accuracy of the risk score in estimating prognosis. All statistical *p*-values were two-sided, and $p < 0.05$ was considered statistically significant.

Supplementary Figure

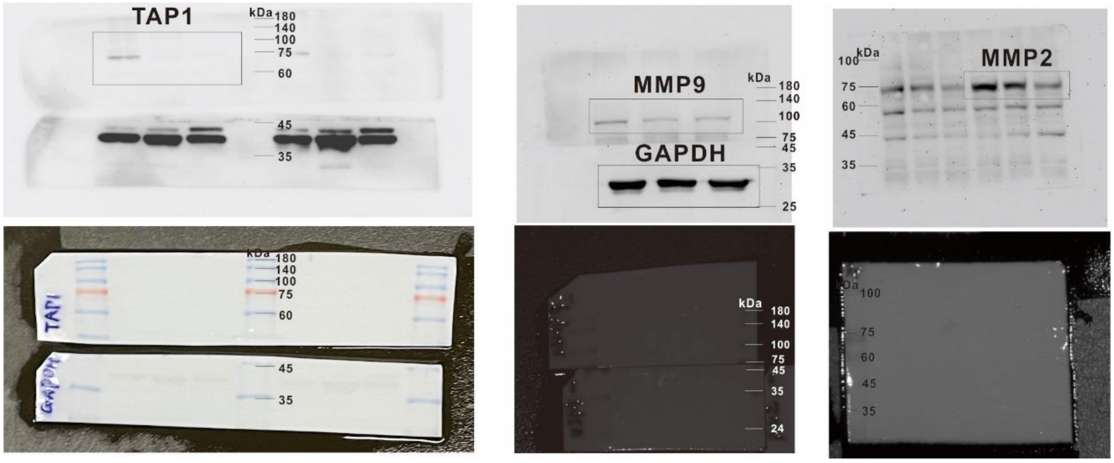

Supplementary Figure S1. Original data of Western Blot

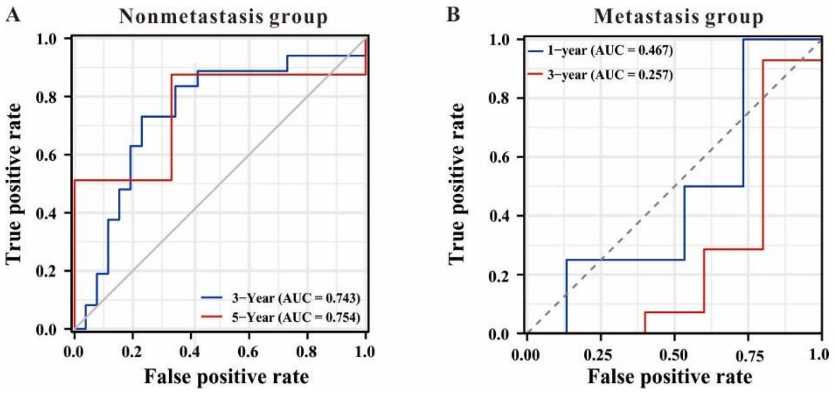

Supplementary Figure S2. ROC curve analysis. **(A)** ROC curve analysis of the predictive accuracy of *TAP1* gene as a prognosis biomarker in non-metastasis group; **(B)** ROC curve analysis of the predictive accuracy of *TAP1* gene as a prognosis biomarker in metastasis group.

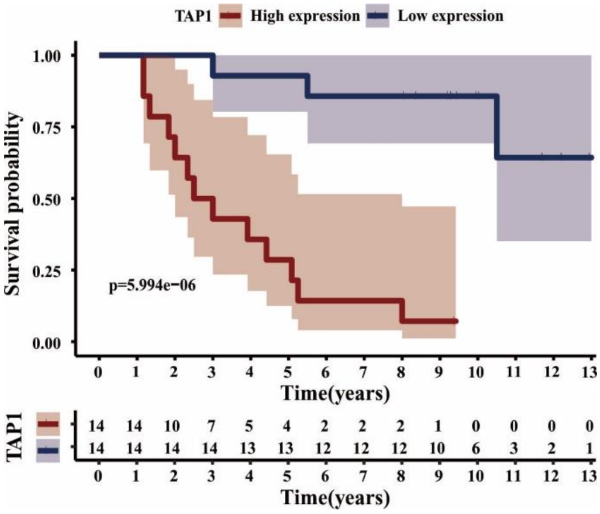

Supplementary Figure S3. Overall survival analysis for GSE84976. Kaplan-Meier analysis for the association between the expression of TAP1 gene with overall survival (OS) in GSE84976 dataset.

Supplementary Table

Supplementary Table S1: siRNA sequence

**Si1:TAP1(H)-729**	
GCCGAUACCUUCACUCGAATT	UUCGAGUGAAGGUAUCGGCTT
**Si2:TAP1(H)-2295**	
CCUGCAGAUGCUCCAGAAUTT	AUUCUGGAGCAUCUGCAGGTT

Supplementary Table S2: Primer sequence

GTTCCTGGTCCTGGTGGTCCTC
GCACTGGCTATGGTGAGAATGGAC

Supplementary Table S3: Cut off value of AUC

Survival Time(year)	AUC	Cut off value	Sensitivity	Specificity	Positive predictive value	Negative predictive value
1	0. 621	3. 194598	0. 794	0. 547	0. 117	0. 972
3	0. 743	3. 713486	0. 731	0. 769	0. 548	0. 882
5	0. 754	2. 317994	0. 875	0. 667	0. 807	0. 770

Reference

1. Laurent C, Valet F, Planque N, Silveri L, Maacha S, Anezo O, Hupe P, Plancher C, Reyes C, Albaud B *et al*: **High PTP4A3 phosphatase expression correlates with metastatic risk in uveal melanoma patients**. *Cancer research* 2011, **71**(3):666-674.
2. van Essen TH, van Pelt SI, Bronkhorst IH, Versluis M, Némati F, Laurent C, Luyten GP, van Hall T, van den Elsen PJ, van der Velden PA *et al*: **Upregulation of HLA Expression in Primary Uveal Melanoma by Infiltrating Leukocytes**. *PloS one* 2016, **11**(10):e0164292.
3. Liu J, Lichtenberg T, Hoadley KA, Poisson LM, Lazar AJ, Cherniack AD, Kovatich AJ, Benz CC, Levine DA, Lee AV *et al*: **An Integrated TCGA Pan-Cancer Clinical Data Resource to Drive High-Quality Survival Outcome Analytics**. *Cell* 2018, **173**(2):400-416.e411.
4. Robin X, Turck N, Hainard A, Tiberti N, Lisacek F, Sanchez JC, Müller M: **pROC: an open-source package for R and S+ to analyze and compare ROC curves**. *BMC bioinformatics* 2011, **12**:77.
